# Relationships between metabolic syndrome and lifestyle factors: a retrospective cohort study in Japan

**DOI:** 10.1016/j.pmedr.2025.103195

**Published:** 2025-08-05

**Authors:** Karin Ueta, Taku Asano, Sachiko Ohde

**Affiliations:** a Graduate School of Public Health, St. Luke's International University, 3-6-2 Tsukiji, Chuo-ku, Tokyo 104-0045, Japan; b Department of Cardiovascular Medicine, St. Luke's International Hospital, 9-1 Akashi-cho, Chuo-ku, Tokyo 104-8560, Japan; c Graduate School of Public Health, Professor of Epidemiology, St. Luke's International University, 3-6-2 Tsukiji, Chuo-ku, Tokyo 104-0045, Japan

**Keywords:** Metabolic syndrome, Lifestyle, Walking, Breakfast, Body mass index, Cohort studies, Survival analysis

## Abstract

**Objective:**

Japan allocates approximately 21.15 billion yen annually for specific health guidance targeting metabolic syndrome (MetS), yet the prevalence of this condition remains unchanged. This study involved identifying lifestyle factors that lower the risk of developing MetS through a comprehensive cohort study encompassing the adult population (i.e., Japanese people aged ≥18 years with no preexisting MetS).

**Methods:**

Data were collected at the St. Luke's International Hospital Clinic Preventive Medical Center in Japan between January 2012 and December 2022. A retrospective cohort study was conducted on the adult population aged 18 years or older who did not develop MetS and underwent health checkups during the study period. Lifestyle factors that were preventively associated with MetS were investigated by using a Cox proportional hazards regression model.

**Results:**

Among the 52,516 included subjects, 5482 (10.4 %) developed MetS. The Cox proportional hazards analysis revealed that a lower risk of developing MetS was associated with walking for >one hour/day (HR = 0.74, 95 % CI = 0.66, 0.83), skipping breakfast <three days/week (HR = 0.86, 95 % CI = 0.76, 0.98), and eating within two hours before bedtime <three days/week (HR = 0.88, 95 % CI = 0.78, 0.99).

**Conclusions:**

In a large-scale cohort study of the adult population in Japan**,** lifestyle factors associated with a lower risk of MetS were walking more than one hour daily, eating breakfast, and not eating before bedtime. Incorporating metabolic indicators in current health guidance may enhance prevention strategies. Further multicenter studies are warranted to support the implementation of national health guidance in Japan.

## Highlights

• This study investigated lifestyle factors that prevent metabolic syndrome.
• This is a large retrospective cohort study involving 52,516 Japanese adults.
• Walking for over an hour daily is the most effective lifestyle factor to prevent metabolic syndrome.
• Eating breakfast is a preventive effect for metabolic syndrome.
• Health guidance to increase metabolism may help prevent metabolic syndrome.

## 1 Introduction

Metabolic syndrome (MetS) has become a global health concern, with a prevalence ranging from 12.5 % to 31.4 % according to various definitions (Noubiap et al., 2022). Notably, in the United States, the prevalence increased to 34.7 % (95 % CI, 33.1, 36.3) between 2011 and 2016, indicating an increasing trend (Hirode and Wong, 2020). The associations between the MetS status and the risk of experiencing adverse health outcomes, including cardiovascular disease (CVD) and mortality, have been extensively investigated, revealing significantly elevated risks of CVD (RR: 2.35; 95 % CI, 2.02, 2.73), CVD mortality (RR: 2.40; 95 % CI, 1.87, 3.08), and all-cause mortality (RR: 1.58; 95 % CI, 1.39, 1.78) in individuals with MetS compared with control participants (Mottillo et al., 2010).

Effective lifestyle modifications, such as aerobic exercise and dietary changes, have shown promise in mitigating MetS, as evidenced by various systematic reviews and meta-analyses of randomized controlled trials (RCTs) demonstrating improvements in biomarkers such as body mass index (BMI), insulin resistance, and visceral fat mass (Kumar et al., 2019; Ostman et al., 2017; Wang et al., 2021; Yarizadeh et al., 2021).

In Japan, significant efforts, backed by a budget of approximately 21.15 billion yen, have been invested in health guidance initiated by the Ministry of Health, Labor and Welfare (MHLW) to combat MetS through lifestyle enhancements (Ministry of Health, Labour and Welfare, Japan, 2021a). However, the effectiveness of these interventions has been questioned, particularly in terms of achieving clinically meaningful weight loss, as reported in a study revealing no association between receiving specific health guidance and experiencing substantial weight loss in men (Fukuma et al., 2020). Alarmingly, despite these initiatives, the number of people with MetS and potential MetS in Japan has continued to increase, with a slowing rate of decline observed from 2016 to 2020 (Ministry of Health, Labour and Welfare, Japan, 2022). Current health guidance targets weight, waist circumference, and behavioral changes as outcomes (Ministry of Health, Labour and Welfare, Japan, 2024), but these findings underscore the need for more effective health guidance strategies.

While some cohort studies in Japan have explored the relationships between lifestyle factors and the risk of developing MetS in specific age groups, such as young and middle-aged individuals, no large-scale cohort studies encompassing the adult population have been conducted. Moreover, the identification of lifestyle factors that lower the risk of developing MetS remains contentious, with conflicting findings reported in existing research. For example, a study targeting middle-aged adults associated weight gain and eating before bedtime with the risk of developing MetS (Tsutatani et al., 2017), whereas another study focusing on young adults linked smoking and rapid eating to the development of MetS (Haruyama et al., 2020). Thus, the aim of this study was to bridge these gaps by comprehensively investigating the lifestyle factors that lower the risk of developing MetS across the adult population through a large-scale cohort analysis.

## 2 Methods

### 2.1 Study design and participants

A retrospective cohort study was conducted. The total sample was 454,386 people who underwent health checkups at the St. Luke's International Hospital Preventive Medicine Center in Chuo Ward, Tokyo, between January 2012 and December 2022.

Chuo Ward is a centrally located urban district in Tokyo, encompassing an area of approximately 10.12 km^2^ with a population of approximately 189,025 people (CHUO CITY, Tokyo, Japan, 2025). Despite its relatively small geographic size, Chuo Ward functions as a major center for commerce and finance. The average annual household income in Chuo Ward exceeds the Tokyo metropolitan average (Ministry of Internal Affairs and Communications, Japan, 2023). The center conducts health checkups for approximately 45,000 individuals annually.

The inclusion criteria included individuals aged 18 years or older who attended their initial medical checkup at the center during the target period. Participants were included if they remained free from MetS at their first visit and underwent a subsequent medical checkup more than ten months apart within the specified target period.

The exclusion criteria included individuals deemed at high risk for coronary artery disease, as determined by a Suita score of 56 points or more (Kinoshita et al., 2018); those actively taking medications for blood pressure, blood sugar, or lipids; those undergoing artificial dialysis; and pregnant women.

Of the 454,386 people who underwent health checkups during the study period, 401,870 were excluded for the following reasons (including duplicates): already diagnosed with MetS at the first visit (*n* = 32,249); the interval between the first and second visits was less than nine months (*n* = 76,005); having high risk with a Suita score of 56 or higher (*n* = 101,66); taking antihypertensive drugs (*n* = 20,724); taking hypoglycemic drugs (*n* = 4716); taking lipid-lowering drugs (*n* = 19,009); lack of data on using or not using medications (antihypertensive drugs, hypoglycemic drugs, or lipid-lowering drugs) (*n* = 331,660); undergoing dialysis (*n* = 98); pregnant women (*n* = 142); lack of BMI data (*n* = 7051); lack of waist circumference data (*n* = 17,956); lack of blood pressure data (*n* = 7063); lack of fasting blood glucose (FBG) data (*n* = 762); lack of triglyceride data (*n* = 741); lack of high-density lipoprotein (HDL) data (n = 741); and lack of lifestyle data (*n* = 27,944). Ultimately, 52,516 individuals were included in the analysis.

### 2.2 Data collection

Data collection involved extracting information from both the health checkup questionnaire and the results of a subsequent health checkup conducted approximately one year later. The parameters used for assessing MetS included the waist circumference, which was measured at mid-waist while standing and exhaling lightly, the BMI, systolic blood pressure (SBP), diastolic blood pressure (DBP), HDL cholesterol levels, triglyceride levels, and FBG levels.

Specifically, lifestyle factor data were obtained from the Health Checkup Questionnaire provided by the MHLW. Information such as age, sex, medical history, medication use, weight, height, BMI, blood liver function parameters, blood renal function parameters, and general blood parameters was collected from the medical examination results.

### 2.3 Outcome assessment

The primary outcome of interest was the time to the development of MetS from the baseline date. MetS was defined according to specific criteria, including a waist circumference of 85 cm or more for men and 90 cm or more for women, plus meeting at least two of the following three criteria: elevated blood pressure, abnormal lipid levels, and high blood sugar levels. These criteria included an SBP ≥130 mmHg and/or DBP ≥85 mmHg, a triglyceride level ≥ 150 mg/dL and/or an HDL cholesterol level < 40 mg/dL, and an FBG level ≥ 110 mg/dL (Ministry of Health, Labour and Welfare, Japan, 2021b). The waist circumference standards for MetS that are set by the National Cholesterol Education Program (NCEP) Adult Treatment Panel III (ATP III) are larger for men than for women (Grundy et al., 2005). However, in Japan, the standard is calculated by converting a visceral fat area of 100 cm^2^ or more into waist circumference, and since women have more subcutaneous fat than men do, the standard is larger for women (Ministry of Health, Labour and Welfare, Japan, 2016). In addition, the FBG standard for MetS in the NCEP ATP III is 100 mg/dL or more, but in Japan, a different standard is used, which is 110 mg/dL or more. This standard is derived from the normal FBG standard in Japan, which states that the early morning FBG level is less than 110 mg/dL and the 2-h value in the 75-g oral glucose tolerance test (OGTT) is 140 mg/dL; however, to diagnose MetS during health checkups, only FBG values, which can be easily measured, have been added to the diagnosis (Ministry of Health, Labour and Welfare, Japan, 2016). In the NCEP ATP III, the HDL criteria for MetS differ between men and women; however, in Japan, according to the serum lipid values that are set by the Japan Atherosclerosis Society for screening of the population and identifying those who require prevention and treatment of arteriosclerosis disease, the diagnostic criterion for MetS is an HDL level of less than 40 mg/dL for both men and women (Ministry of Health, Labour and Welfare, Japan, 2016).

### 2.4 Covariates

In analyses, we controlled for the following variables: Age, Sex, BMI, Uric acid level, RBC count, Walk an hour a day, Dinner before sleeping, Skip breakfast, Walking speed, Eating speed, Frequency of eating out for dinner, and Alcohol consumption frequency.

These covariates were chosen as follows. Sex was selected because there are many men with MetS in Japan (Ministry of Health, Labour and Welfare, Japan, 2019). The BMI, an indicator of obesity, was selected because obesity is related to MetS (Krishnamoorthy et al., 2022; Noubiap et al., 2022; Tsutatani et al., 2017). Similar previous studies in Japan have shown associations between MetS and low physical activity, having dinner before sleep, an eating speed, and drinking alcohol (Haruyama et al., 2020; Tsutatani et al., 2017). Therefore, we selected Walk an hour a day, Dinner before sleeping, Walking speed, Eating speed, Frequency of eating out for dinner, and Alcohol consumption frequency as covariates. Among the variables related to activity, we selected as covariates only Walk an hour a day and Walking speed, which were particularly significant. Uric acid level, RBC count, and Skip breakfast were not listed as potential covariates but were added because they were highly significant.

Test parameters were collected from height and weight, measured on the health checkup day, and blood samples were taken. Lifestyle factors were assessed by using a standard questionnaire from the Japanese MHLW (Ministry of Health, Labour and Welfare, Japan, 2023), which was used at the target centers. This questionnaire was mailed to all patients in advance, and they were asked to fill it out at home and bring it with them. If any information was omitted, people were asked to fill it out during the reception or medical interview. Lifestyle questions regarding diet, exercise, sleep, smoking, and alcohol consumption were collected.

### 2.5 Statistical analysis

**Fig. 1:**
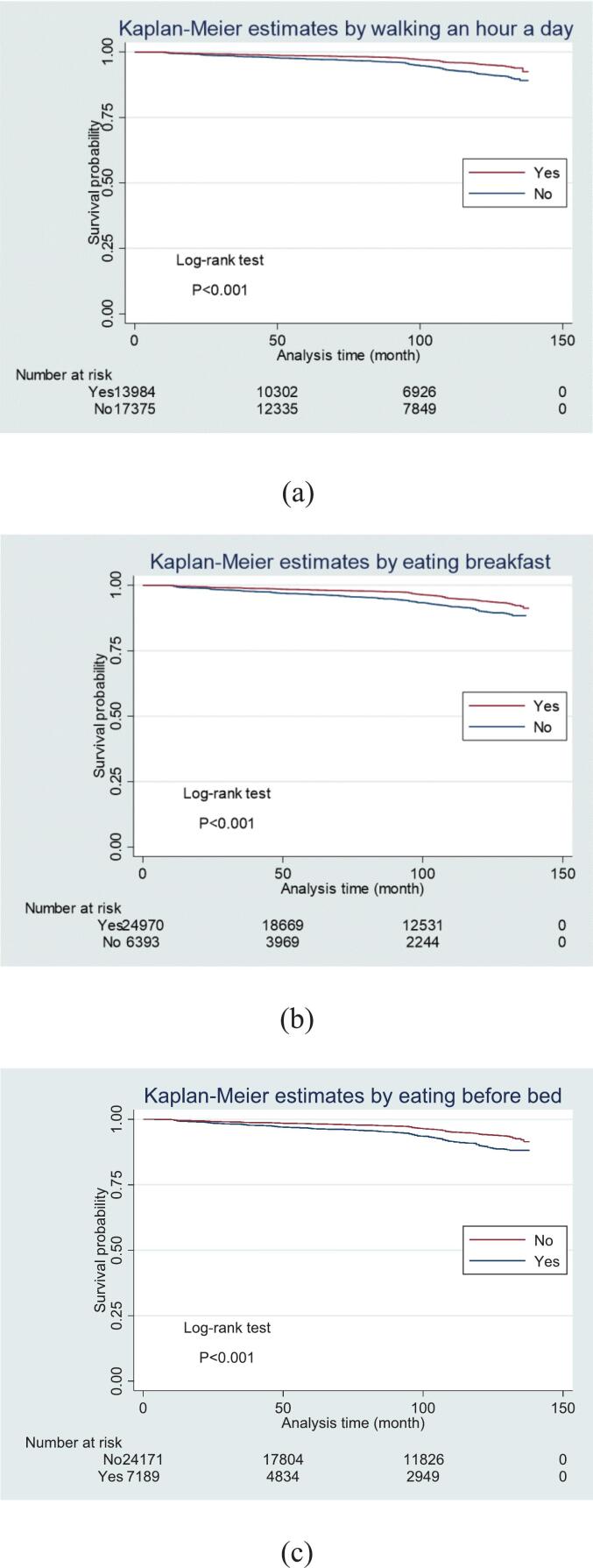
Survival analysis of various factors' association with metabolic syndrome incidence from St. Luke's International Hospital in Tokyo, Japan (2012–2022). Notes: (a) Walking an hour a day, (b) Eating breakfast, (c) Eating before bedtime. (a) Yes = people who walk for more than one hour a day. No = people who do not walk for more than one hour a day. (b) Yes = people who skip breakfast less than three days a week. No = people who skip breakfast three or more days a week. (c) Yes = people who eat before bedtime three or more days a week. No = people who eat before bedtime less than three days a week.

The baseline characteristics of the participants from the MetS and non-MetS groups were summarized (Table 1). Continuous variables were presented as means and standard deviations (SD) and analyzed using independent *t-*tests, while categorical variables were reported as frequencies with percentages and compared via chi-square tests. Kaplan-Meier survival analysis (Fig. 1) was conducted to explore the associations between lifestyle factors and the incidence of MetS, with survival differences evaluated using log-rank tests. Cox proportional hazards models were applied for univariate (Table S1) and multivariable (Table 2) analyses to estimate HR and 95 % CI for determining the impact of lifestyle factors on MetS development. Given the large sample size, a stringent significance level of *p* < 0.001 was adopted for univariate analyses to reduce the likelihood of detecting statistically significant yet clinically negligible associations. A conventional threshold of *p* < 0.05 was applied for multivariable analyses, where independent associations were evaluated after adjusting for confounding factors. Survival time was defined as the interval from the initial health checkup to the onset of MetS or censoring events, such as loss to follow-up or death. The statistical software STATA SE-17 (64-bit; College Station, Texas, USA) was used for all analyses.

**Table 1:** Characteristics of adult participants by metabolic syndrome status from St. Luke's International Hospital in Tokyo, Japan (2012−2022).

Characteristics		MetS	No MetS	*p* value
Age, mean (SD)		52.1 (11.6)	52.2 (13.3)	0.614
Sex, N (%)	Women	1230 (4.2)	28,419 (95.9)	<0.001
	Men	4252 (18.6)	18,615 (81.4)	
BMI, mean (SD)		25.9 (2.6)	21.7 (2.9)	<0.001
Uric acid level, mean (SD)		6.3 (1.3)	5.3(1.3)	<0.001
RBC count, mean (SD)		4.80 (0.42)	4.49 (0.43)	<0.001
Walk an hour a day, N (%)	Yes	430 (3.1)	13,556 (96.9)	<0.001
	No	888 (5.1)	16,491 (94.9)	
Dinner before sleeping, N (%)	Yes	428 (6.0)	6762 (94.1)	<0.001
	No	892 (3.7)	23,284 (96.3)	
Skip breakfast, N (%)	Yes	344 (5.8)	6052 (94.6)	<0.001
	No	976 (3.9)	23,997 (96.1)	
Walking speed, N (%)	Fast	721 (3.8)	18,078 (96.2)	<0.001
	Slow	599 (4.8)	11,964 (95.2)	
Eating speed, N (%)	Fast	1992 (12.2)	14,350 (87.8)	<0.001
	Somewhat fast	1527 (21.9)	5433 (78.1)	
	Normal	1655 (6.9)	22,226 (93.1)	
	Slow	292 (5.6)	4926 (94.4)	
Frequency of eating out for dinner, N (%)	5 days or more/week	608 (21.6)	2210 (78.4)	<0.001
	3–4 days/week	1019 (16.6)	5118 (83.4)	
	1–2 days/week	2138 (10.3)	18,365 (89.6)	
	Almost never	1700 (7.41)	21,241 (92.6)	
Alcohol consumption frequency, N (%)	Do not consume	1247 (15.3)	6909 (84.7)	<0.001
	Hardly	562 (3.8)	14,378 (96.2)	
	Sometimes	530 (19.4)	2205 (80.6)	
	Often	902 (5.02)	17,068 (95.0)	
	Habitually	2240 (25.7)	6470 (74.3)	

Notes: *P*-values are based on t-tests or chi-square tests, as appropriate.

Abbreviations: N, number; SD, standard deviation; BMI, body mass index; RBC, red blood cell.

**Table 2:** Multivariable analysis of the association of various factors with the incidence of metabolic syndrome in adults from St. Luke's International Hospital in Tokyo, Japan (2012–2022).

Variables	Adjusted HR	95 % CI	
		Lower	Upper
Walk an hour a day	0.74	0.66	0.83
Dinner before sleeping	0.88	0.78	0.99
Skip breakfast	0.86	0.76	0.98
Walking speed	0.94	0.84	1.06
Eating speed	1.01	0.95	1.06
Frequency of eating out for dinner	1.07	0.99	1.14

Notes: Hazard ratios were adjusted for the following covariates:

age, sex, body mass index (BMI), uric acid level, red blood cell (RBC) count, and alcohol consumption frequency.

Abbreviations: HR, hazard ratio; 95 % CI, 95 % confidence interval.

### 2.6 Ethics statement

This was an opt-out study, and patients were provided with information about the study and were guaranteed the right to refuse to participate, in accordance with Japan's “Ethical Guidelines for Medical Research Involving Human Subjects.” The protocol was submitted to the hospitals that provided data to ensure the anonymization of the data and subsequently received approval from the responsible personnel. The data handling processes were exclusively managed by the researcher and were performed in a controlled environment, either at St. Luke's International University Graduate School or at the researcher's home. Stringent security measures, including password protection on personal computers, were implemented to safeguard the data. Ethical approval for this research was obtained from St. Luke's Ethics Review Committee (approval code: 23-R041).

## 3 Results

### 3.1 Characteristics of the participants

The study included 52,516 subjects, with 5482 (10.4 %) developing MetS. Within this cohort, 4.2 % of women and 18.6 % of men developed MetS. The mean age was 52.1 years in the MetS group and 52.2 years in the non-MetS group, with a mean BMI of 25.9 kg/m^2^ in the MetS group and 21.7 kg/m^2^ in the non-MetS group (Table 1). The median time to the onset of MetS during the observation period was not available.

### 3.2 Factors related to the onset of MetS according to Kaplan–Meier curves

The Kaplan-Meier curves revealed that walking more than one hour a day (Fig. 1a), skipping breakfast less than three days a week (Fig. 1b), and eating within two hours before bedtime less than three days a week (Fig. 1c) were associated with lower HR for incident MetS (log-rank test, *p* < 0.001).

### 3.3 Lifestyle factors and parameters associated with the development of MetS identified using cox proportional hazards models

In this extensive cohort study encompassing the adult population, the Cox proportional hazards model revealed that the lifestyle factor with the lowest HR was walking for an hour a day (HR = 0.74, 95 % CI = 0.66, 0.83), followed by skipping breakfast less than three times a week (HR = 0.86, 95 % CI = 0.76, 0.98) and eating dinner within two hours before going to bed less than three times a week (HR = 0.88, 95 % CI = 0.78, 0.99) (Table 2).

## 4 Discussion

This comprehensive study of the adult population in Japan found that the lifestyle factors that lowered the risk of developing MetS were walking for more than one hour a day, skipping breakfast less than three days a week, and eating within two hours before going to bed less than three days a week.

Consistent with the findings of previous studies (Kikuchi et al., 2021; Ministry of Health, Labour and Welfare, Japan, 2019), our findings reaffirm the higher prevalence of MetS among men in Japan. The associations of MetS with a higher BMI and alcohol consumption and the association of a lower risk of developing MetS with light exercise that were observed in the present study align with the findings of previous studies (Haruyama et al., 2020; Kikuchi et al., 2021; Kobo et al., 2019). However, our study diverges from prior research by highlighting unique lifestyle factors, such as walking for more than an hour a day and not skipping breakfast, that were found to be associated with a lower risk of developing MetS. These lifestyle factors, which are relatively easy to incorporate into daily life, hold promise for preventing MetS with potential long-term adherence.

Although the energy intake of Japanese people is decreasing (Ministry of Health, Labour and Welfare, Japan, 2021c), the incidence of MetS is increasing (Ministry of Health, Labour and Welfare, Japan, 2022). A scoping review of observational studies examining the relationship between high protein intake at breakfast and increased muscle mass revealed that approximately 58.8 % of the 11 studies reported that participants had increased muscle mass (Khaing et al., 2025). Eating protein at breakfast is also associated with more significant muscle mass gain than eating protein at other meals (Kim et al., 2021). This means that to reduce MetS, it may be essential to not only reduce the body weight and waist circumference but also to increase basal metabolism by increasing muscle mass through the consumption of protein at breakfast and engaging in an appropriate walking routine. This result suggests the need to add outcomes that measure metabolism, such as muscle mass, to the health guidance that uses only the current weight, waist circumference, and behavioral changes as outcomes (Ministry of Health, Labour and Welfare, Japan, 2024). A previous study (Fukuma et al., 2020) showed that specific health guidance for men in Japan was not associated with weight loss, whereas this study showed that these lifestyle factors were associated with a lower risk of developing MetS, even after adjusting for sex. By providing specific health guidance focusing on these lifestyle factors, it may be possible to reduce MetS risks, even in Japan, where its prevalence is high among men.

Despite its strengths, this study has several limitations. Self-reported lifestyle factors may introduce bias, and data from a single center may limit generalizability. The prevalence of MetS in Japan is estimated to be 17.8 % (Ministry of Health, Labour and Welfare, Japan, 2019); however, the incidence rate of MetS in this study was very low, at 10.4 %. This study was conducted at a single facility in Tokyo, an urban area with high household incomes in Japan, which may have influenced the results and led to an underestimation of the MetS incidence. Considering this low incidence rate of MetS, the follow-up period for the evaluation at the health checkup, approximately one year later, may have been short, and future studies should set a longer evaluation interval, such as three or five years. Potential confounding factors may exist because of variables not present in the questionnaire or the method of extracting the independent variables. Moreover, further multicenter studies will be necessary to increase the applicability of these findings to national health guidance.

## 5 Conclusions

Through a large-scale cohort analysis of the adult population, lifestyle factors associated with a lower risk of developing MetS were found to be walking for more than one hour per day, skipping breakfast less than three days per week, and eating within two hours before going to bed less than three days per week. The results of this study highlight the need for effective and targeted interventions in Japan, where specific health guidance for MetS consumes a significant portion of the national budget. While current health guidance indicators in Japan primarily include body weight, waist circumference, and behavioral changes, incorporating metabolic indicators, such as muscle mass, in future evaluations may yield novel insights and contribute to more comprehensive preventive strategies. Future multicenter studies will be needed to generalize these observed results to health guidance in Japan.

## CRediT authorship contribution statement

**Karin Ueta:** Writing – review & editing, Writing – original draft, Visualization, Validation, Software, Project administration, Methodology, Investigation, Formal analysis, Data curation, Conceptualization. **Taku Asano:** Writing – review & editing, Supervision. **Sachiko Ohde:** Writing – review & editing, Validation, Supervision, Software, Project administration, Methodology, Formal analysis.

## Informed consent

This was an opt-out study, and patients were provided with information about the study and were guaranteed the right to refuse to participate, in accordance with Japan's “Ethical Guidelines for Medical Research Involving Human Subjects.” The study was approved by the St. Luke's Ethics Review Committee (23-R041, 10/7/2023).

## Declaration of generative AI and AI-assisted technologies in the writing process

This paper does not use AI.

## Funding

This research did not receive any specific grant from funding agencies in the public, commercial, or not-for-profit sectors.

## Declaration of competing interest

The authors declare that they have no known competing financial interests or personal relationships that could have appeared to influence the work reported in this paper.

## Data Availability

The data that has been used is confidential.

## Appendix A Supplementary data

Supplementary TableS1 Image 1
